# Liquid-Diet with Alcohol Alters Maternal, Fetal and Placental Weights and the Expression of Molecules Involved in Integrin Signaling in the Fetal Cerebral Cortex

**DOI:** 10.3390/ijerph7114023

**Published:** 2010-11-17

**Authors:** Ujjwal K. Rout, Julie M. Dhossche

**Affiliations:** 1 Departments of Surgery, Physiology and Biophysics, University of Mississippi Medical Center, 2500 North State Street, Jackson, MS 39216, USA; 2 University of Mississippi, 1848 University Circle, University, MS 38677, USA; E-Mail: jmdhossche@olemiss.edu

**Keywords:** prenatal, alcohol, weight, fetus, brain, cerebral cortex, integrins

## Abstract

Maternal alcohol consumption during pregnancy causes wide range of behavioral and structural deficits in children, commonly known as Fetal Alcohol Syndrome (FAS). Children with FAS may suffer behavioral deficits in the absence of obvious malformations. In rodents, the exposure to alcohol during gestation changes brain structures and weights of offspring. The mechanism of FAS is not completely understood. In the present study, an established rat (Long-Evans) model of FAS was used. The litter size and the weights of mothers, fetuses and placentas were examined on gestation days 18 or 20. On gestation day 18, the effects of chronic alcohol on the expression levels of integrin receptor subunits, phospholipase-Cγ and N-cadherin were examined in the fetal cerebral cortices. Presence of alcohol in the liquid-diet reduced the consumption and decreased weights of mothers and fetuses but increased the placental weights. Expression levels of β_1_ and α_3_ integrin subunits and phospholipase-Cγ_2_ were significantly altered in the fetal cerebral cortices of mothers on alcohol containing diet. Results show that alcohol consumption during pregnancy even with protein, mineral and vitamin enriched diet may affect maternal and fetal health, and alter integrin receptor signaling pathways in the fetal cerebral cortex disturbing the development of fetal brains.

## 1. Introduction

Alcohol consumption during pregnancy disturbs the normal development of fetus causing wide range of structural and behavioral problems in children commonly known as fetal alcohol syndrome (FAS) [1]. Reduced birth weight and mental deficiencies are the hallmarks of FAS that persists even in adolescence [2]. Low birth weight is estimated to be about 10% of health care costs for children [3] and is linked to obesity [4], hypertension, diabetes mellitus [5], and reduced fertility [6] later in life. FAS is considered to be the leading cause of mental retardation in the Western world [7].

Normal functioning of brain requires optimum migration of neurons in the fetal brains [8,9]. Several studies with Long-Evans rats now show that the migration and proliferation of fetal cerebral cortical neurons is altered by prenatal alcohol even when the alcohol in the protein- and nutrient-enriched liquid-diet is increased in a gradual and step-wise manner [10–13]. *In vitro* studies with slice culture of fetal cerebral cortex from Sprague-Dawley rats reveal that alcohol may alter the expression of integrin subunits that are involved in the migration of neurons [14]. A recent study shows that acute alcohol exposure during early gestation alters transcript levels of integrin subunits and extra cellular matrix (ECM) proteins in the fetal head fold of mouse in a genotype dependent manner [15]. Yet, the effects of prenatal alcohol on maternal diet consumption, and the weights of mother, fetus and placenta in Long-Evans rats, a species extensively used for studying effects of maternal alcohol on fetal brains, are not known. Moreover, the effects of chronic prenatal (*in vivo*) alcohol on the expression levels of molecules involved in the integrin signaling in the fetal cerebral cortex are currently not known. Therefore, in the present study an established rat model of FAS was used to examine the effects of maternal alcohol on the body weights of mothers and fetuses as well as on the expression levels of molecules involved in the integrin signaling in the fetal cerebral cortex.

## 2. Materials and Methods

### 2.1. Animal Compliance

All procedures performed on rats, including the use of diet regimens for the studies, were according to an animal protocol approved by the University of Mississippi Animal Care and Use Committee.

### 2.2. Animals and Alcohol Exposure

Pregnant Long-Evans rats were obtained from Charles River (MA). Animals were acclimated on regular chow and water in the laboratory animal facility of The University of Mississippi Medical Center in a 12 h light (6 AM to 6 PM) and dark cycle. On gestation day (GD) 5, animals were weighed and divided into control and experimental groups each consisting of animals of similar weight range with average weight of 256 g. Each group consisted of total 8 pregnant mothers. Starting on gestation day 6 morning, food and water supplies were replaced with 110 mL liquid-diet F1265SP (Bio-SERV, French Town, NJ, USA) in graduated glass feeding tubes. F1265SP is a Lieber-DeCarli diet [16] enriched with proteins, vitamins and minerals. The components of this diet included, Corn Syrup, Casein, Olive Oil, Maltodextrin, Cellulose, Mineral Mix, Corn Oil, Suspending Aids, Safflower Oil, Vitamin Mix, L-Cystine, Choline Bitartrate and DL-Methionine.

Alcohol (ethanol) was added into the diet for the experimental groups of animals. Maltose-dextrin was added into the control diet to ensure that the control and alcohol-containing diets were isocaloric. Concentration of alcohol (v/v) in the diet was increased with the gestation and maintained at 2.2% during GD 6 & 7, 4.5% during GD 8, 9 & 10, and 6.7% during GD 11 to 17 or 19 according to a previously published protocol [10] that has been used to examine the toxic effects of maternal alcohol on fetal brains. Because in this protocol significant differences in the migration of only late generated neurons were recorded between the pair-fed (Chow and water) and control Liquid-Diet (minus alcohol) groups, the pair-fed group was not included in our study. In addition, the Chow and water group of control was excluded from our study because no differences in the results between this group and liquid diet with no alcohol group is reported in other studies [17,18], The volumes (mL) of diet consumed every 24 h by individual rats were recorded in the mornings (9 AM) and freshly prepared diets were supplied in clean tubes.

### 2.3. Maternal Weights and Blood Alcohol Concentrations

On GD18 or GD20 morning (9 AM) animals were anesthetized with isoflurane (Abbott Labs., Chicago, IL, USA) and weighed. The abdomen was opened with a pair of scissors, and the diaphragm ruptured for the collection of blood from the heart with a syringe. Blood (0.5 mL) was transferred into an anticoagulant blood collection tube. Alcohol concentration in the blood (mg/dL) was determined by head space gas chromatography.

### 2.4. Fetal and Placental Weights, and Gross Morphology

The uteri were dissected out and transferred into a tray containing chilled phosphate buffer saline (PBS). Fetuses and placentas were collected from uteri and transferred into plastic Petri dishes containing chilled PBS. Fetuses and placentas were separated with a pair of scissors, soaked with facial tissue papers to remove extra PBS and weighed. Number of fetuses per mother was recorded. General morphology of fetuses (such as presence of cleft) and placentas were examined visually.

### 2.5. Isolation of Fetal Cerebral Cortices and Western Blotting

Heads from gestation day 18 fetuses were transferred into chilled neuron culture medium DMEM/F12 (Invitrogen, Carlsbad, CA, USA) for the removal of brains. Cerebral cortices were dissected out and meninges removed (Figure 4A). Cerebral cortices from 2 fetuses per mother were pooled and subjected to homogenization in the TPER solution containing Halt proteinase inhibitor (Thermo Fisher Scientific, Inc., Rockford, IL, USA). The homogenates were centrifuged at 90 g and 4 °C for 10 min. The clear supernatants were stored at −20 °C. Aliquots of supernatants were subjected to protein assays using a BCA protein estimation kit (Thermo Fisher Scientific) as described earlier [19]. Fetal cerebral cortical proteins (15 μg) from control (liquid-diet only) and alcohol (liquid-diet plus alcohol) exposed mothers were subjected to polyacrilamide (10%) gel electrophoresis and Western blotting as described earlier [19]. Membranes were exposed to primary antibodies (Table 1) in blocking solution overnight at 4 °C, Membranes were washed and exposed to peroxidase-conjugated secondary antibodies (Jackson Immunoresearch, West Grove, PA, USA) (100 ng/mL PBS) for 1h at room temperature. Bands representing respective antigens were detected using a Chemiluminescent detection system and Amersham Hyper film ECL (Both from GE Healthcare Ltd., Buckinghamshire, UK). Optical densities of bands were determined using ImageJ Software as described earlier [19].

### 2.6. Statistical Analysis

SPSS software (SPSS Inc., Chicago, IL, USA) was used to determine the mean and the standard errors of mean of diet volume; maternal, fetal, placental weights and optical densities of bands. ANOVA (Post hoc analysis) was conducted to determine the significance of differences of mean + standard error of mean values obtained from control and alcohol-exposed animals at *p* < 0.05.

## 3. Results

### 3.1. Diet Consumption

**Figure 1:**
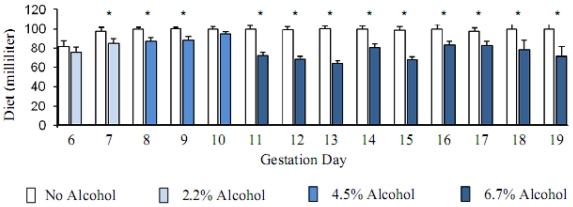
Alcohol reduced the consumption of liquid-diet. Mean + Standard error of mean of diet volume (milliliter) consumed by the pregnant rats starting gestation day 6. Alcohol in the diet reduced the consumption significantly (* *p* < 0.05).

**Figure 2:**
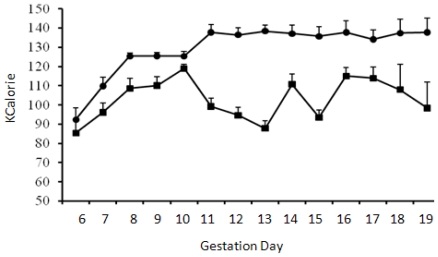
Calories ingested by pregnant animals. Mean + standard error of mean of calories consumed by the pregnant rats starting gestation day 6. Calories consumed by the control (●) and alcohol (■) groups of animals were significantly different (*p* < 0.05) starting GD11.

Pregnant animals consumed ~100 mL control liquid-diet during the whole course of diet regimen except the first day (GD6) of transition from the diet containing chow and water into the liquid-diet regimen, when the consumption was less (~82 mL). Volume of liquid-diet containing 2.2% alcohol was similar to the control diet (p > 0.05) on the first day of liquid-diet regimen. Afterwards and throughout the diet regimen, the consumption of alcohol-containing diet mostly remained significantly lower (p < 0.05) than the control diet. Consumption of the diet with 2.2% alcohol during GD7 was 13% lower than the controls; whereas consumption of the diet with 4.5% alcohol during GD8-GD10 was 8–13% lower than the control diet. When the alcohol concentration in the diet was raised to 6.7% during GD11 to GD19, consumption of the diet dropped even further and was 11–30% lower than the control diet. The total volume of the alcohol-containing diet consumed by the pregnant rats during gestation days 6 to 19 was about 17% lower than the control diet. Volumes of the diets consumed by the control and alcohol groups of pregnant animals are shown below (Figure 1). The calories ingested by the control and alcohol groups of pregnant animals are shown in Figure 2.

### 3.2. Blood Alcohol Concentration

The mean blood alcohol concentration in mothers at GD18 (n = 8) and GD20 (n = 8) were ~49 and 61 mg/dL, respectively. The maximum blood alcohol levels were 126 and 155 mg/dL on GD18 and GD20, respectively.

### 3.3. Alcohol Effects on Weights of Mothers, Fetuses and Placentas

**Figure 3:**
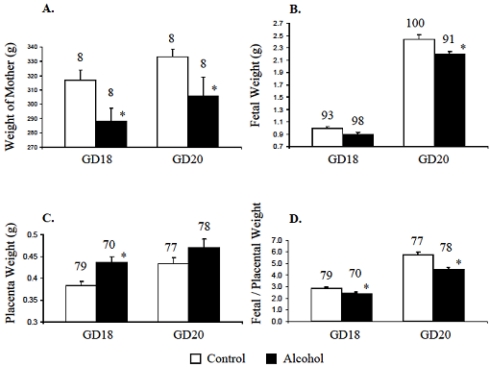
Prenatal alcohol changed maternal, fetal and placental weights. A. Alcohol in diet reduced the weights of pregnant mothers on gestation days 18 and 20. B. On gestation days 18 and 20, the weights of fetuses from mothers on the alcohol diet were also lower than those of fetuses from mothers on the control diet. C. Alcohol in diet increased the placenta weights. D. The fetal/placental ratios on gestation days 18 and 20 were reduced due to maternal alcohol. Total number of mother, fetuses and placenta are mentioned on top of respective bar. * Significantly different from respective control at *p* < 0.05.

Weights of pregnant mothers on alcohol were significantly lower (*p* < 0.05) than the weights of those on control diet (Figure 3A). Weights of fetuses at GD18 and GD20 from mothers on the alcohol diet were also lower compared to those from mothers on the control diet at GD18 and GD20 respectively (Figure 3B). Prenatal alcohol increased the weights of placentas isolated from GD18 and GD20 mothers (Figure 3C). Accordingly, the fetal/placental weight ratio at GD18 and GD20 decreased due to chronic alcohol ingestion during gestation (Figure 3D).

### 3.4. Litter Size and Morphology

The number of fetuses from mothers on control or alcohol containing diet varied from 12 to 14, and the means + standard error of mean values of fetal numbers were not significantly different between the two groups. All fetuses born from control and alcohol fed mothers were alive. No obvious differences in facial features (such as cleft or abnormal eye), limbs, tail and body were found between the control and alcohol-exposed fetuses at GD18 and GD20. Placentas from the control and alcohol treated groups had no gross morphological differences.

### 3.5. Expression Levels of Molecules in the Fetal Cerebral Cortex

**Figure 4:**
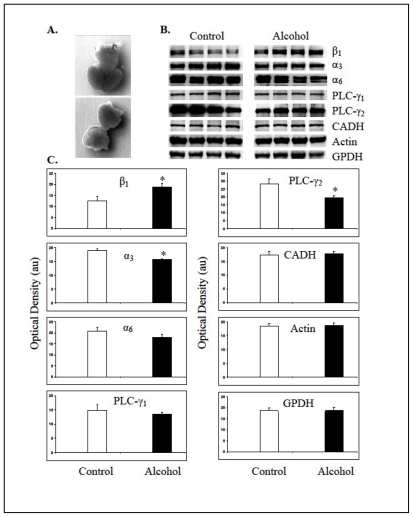
Maternal alcohol changed expression levels of molecules involved in integrin signaling in the fetal cerebral cortices. A. Brain from a GD18 fetal head (top) and dissected cerebral cortices (bottom). B. Bands representing expression levels of integrin subunits (β_1_, α_3_ and α_6_), phospholipase Cγ isoforms (PLC-γ_1_ and PLC-γ_2_), N-cadherin (CADH), β-Actin and GPDH from control and alcohol exposed fetal cerebral cortices. Each band represents expression of molecule in a pooled sample of cortices from two fetuses per mother. C. Mean + standard errors of mean values of the optical densities of bands in arbitrary units (au) from control and alcohol exposed cerebral cortices. * Significantly different from the respective controls at p < 0.05.

Expected molecular weight bands representing β_1_ (130 kDa), α_3_ (135 kDa), PLC-γ_1_ (155 kDa), PLC-γ_2_ (155 kDa), N-cadherin (140 kDa), β-actin (42 kDa) and GPDH (36 kDa) were detected in the fetal cerebral cortices using the Western blotting technique (Figure 4B). The antibody against α_6_ integrin subunit recognized two electrophoresis bands from the fetal cerebral cortex, an expected 127 kDa band and an additional band at 150 kDa, which may represent two splice variants of this subunit [20]. Analysis of band intensities revealed that gestational alcohol increased the expression of β_1_ integrin subunit in the cerebral cortex of GD18 fetuses. Contrarily, the expression level of α_3_ integrin subunit was decreased significantly (*p* < 0.05). The expression level of α_6_ integrin subunit was also decreased in the fetal cerebral cortices exposed to alcohol; however this decrease was not statistically significant (*p* > 0.05). The expression levels of phospholipase C (PLC)- γ_1_ were not different between the control and alcohol exposed cortices and the PLC-γ_1_ was not phosphorylated at tyrosine 783 (data not shown). The authenticity of Phospho-PLC-γ_1_ (Tyr 783) antibody was verified in a cancer cell line (data communicated elsewhere). Remarkably high levels of PLC-γ_2_ were detected in the fetal cortices that decreased significantly (*p* < 0.05) due to prenatal alcohol exposure (Figure 4B and Figure 4C). The expression levels of N-cadherin, β-actin and GPDH were not significantly different between control and alcohol-exposed groups of fetal cortices.

## 4. Discussion

Results presented here show for the first time that (i) the pregnant Long-Evans rats consumed significantly less of a protein fortified liquid-diet (F1265SP) when the alcohol in the diet is increased in a step-wise manner and (ii) that in this species of rat, the expression of molecules involved in the integrin signaling are significantly altered in the cerebral cortices of fetuses exposed to alcohol during gestation even in the absence of obvious morphological defects in the offspring. These results show that the pregnant Long-Evans rats on the liquid-diet containing alcohol are undernourished and that the fetal cerebral cortical development may be impaired due to disturbances in integrin receptor signaling.

High protein concentration or step-wise increase of alcohol in the liquid-diet is known to increase diet consumption and maintain weights of pregnant Sprague-Dawley rats [17,18]. Therefore, the suboptimal consumption of liquid-diet enriched with proteins, minerals and vitamins by the pregnant Long-Evans rats, even when alcohol in the diet was increased in a step-wise manner, suggests that the consumption of liquid-diet with alcohol—and thus the outcomes of prenatal alcohol—may differ between strains of rats. Maternal genotype [21], genetic differences in the extent of taste aversion for alcohol in the diet [22] or an increase in the sleep-time caused by the alcohol [23] in these strains may determine the extent of maternal alcohol consumption during pregnancy. Genetics may also determine the effects of undernourishment on the mother and offspring. More severe reduction in maternal weight gain, fetal body weight and increased placenta weights are associated with dietary restrictions in genetically distinct C57BL/6J *versus* A/J mouse strains [24,25]. Moreover, in the context of the proposed role of maternal genotype on the outcome of dietary restriction in children [26], the present findings suggests that the Long-Evans rats may be genetically susceptible for the malnutrition-induced loss of body weight.

A single intra-peritoneal injection of high alcohol dose during gestation reduces fetal weights in C57BL/6J mice [27], and alcohol in the diet is shown to reduce intrauterine growth by suppressing insulin-like growth factor-1 and reducing the transfer of 2-deoxy glucose to the fetus via the placenta [18,28]. On the other hand, malnutrition itself may affect the weight of offspring by reducing Insulin like growth factor expression [29], and by increasing alcohol-induced feto-toxicity mediated by the reduced liver alcohol and aldehyde metabolizing enzymes in the fetus [30,31]. Therefore, both the undernourishment caused by the decreased consumption of liquid-diet containing alcohol as well as the direct effects of alcohol may be the contributing factors in reducing the weights of pregnant Long-Evans rats.

Increased placental weights and the decreased fetus/placenta ratios (Figure 3) observed in the alcohol-exposed mothers were also found in earlier studies with Wistar, Sprague-Dawley, and albino rats [32–35]. So the increase in placental weights in rats on alcohol-containing diet may be a common phenomenon irrespective of the strains. Placental weight is also reported to increase due to malnutrition itself; and this concurrence is interpreted as a compensatory mechanism to reduce the effects of maternal under-nutrition on the fetus [36].

The lack of difference between the litter size of the control and alcohol fed groups and the absence of dead fetuses in this study, as opposed to previous studies [37,38], may in part be due to the low concentrations of alcohol in the diet during the first trimester. This may also be due to the delayed start of alcohol liquid-diet regimen, from GD6, in this study as opposed to the time of conception in other studies [37,39], which may reduce embryo implantation and cause fetal resorption decreasing the litter size in alcohol-exposed mothers.

Even though visual examination of the fetuses from Long-Evans rats on alcohol showed no gross morphological changes, results of Western blotting studies revealed that the prenatal alcohol in the liquid-diet influenced molecules that are directly or indirectly involved in the integrin receptor signaling. This included heightened expression of integrin subunit β_1_ (*p* < 0.05) and decreased expression of α_3_ subunit (*p* < 0.05), that are required to form the functional β_1_α_3_ integrin receptor for the migration of neurons in fetal brains [40]. In previous studies, high concentrations of ethanol (400 mg/dL) in culture is shown to increase the expression level of β_1_ integrin subunit in B104 neuroblastoma cells [41] and in cerebral cortical slices of fetal (GD 17) Sprague-Dawley rats [14]. The latter study also showed elevated levels of α_3_ integrin subunit in the cortical slices cultured in presence of high concentration of alcohol for 6h but not on 12 h. This discrepancy in the α_3_ integrin subunit expression data between the previous [14] and our *in vivo* study could be caused by differences in the concentration of alcohol and treatment times (6 and 12 h) used in the *in vitro* study and those attained in the blood of mother during chronic exposure to alcohol. Indeed, an *in vivo* study shows that acute alcohol intoxication on gestation day 8 lowers α_3_ integrin subunit transcript levels in the head fold of a substrain of C57BL/6 mice [15]. This later study also confirmed that the integrin receptor subunits and ECM molecules in the fetal brains are the direct target of alcohol, *i.e.* without malnutrition as a confounding factor. Nevertheless, all these studies emphasize that alcohol-induced change in the expression levels of molecules in the fetal brains varies with the strains of rodents as well as with the time, dose and pattern of alcohol exposure.

Phosphoinositide-specific phospholipase C (PLC) isoforms play a critical role in mediating integrin receptor signaling by hydrolyzing phosphatidylinositol 4,5-biphosphate (PIP2) to generate second messengers, inositol 1,4,5-triphosphate (IP3) and diacylglycerol (DAG) [42]. Of several PLC isoforms, the PLC-γ_1_ is known to play significant role in integrin signaling and is activated by the epidermal and platelet derived growth factors at tyrosine 771, 783 and 1,245 positions [43]. Data presented here show that the prenatal alcohol neither alters the expression levels of PLC-γ_1_ nor activates it by phosphorylation of tyrosine at position 783. Instead, the prenatal alcohol decreases the expression levels of PLC-γ_2_ isoform in the cerebral cortices, an isoform that is known to influence integrin signaling in osteoclast [44]. These results indicate that prenatal alcohol may alter integrin mediated signaling and functions in the developing cerebral cortices by altering the expression of β_1_ and α_3_ integrin subunits, and PLC-γ_2_. Results from this study also show that the expression levels of N-cadherin, the activity of which is regulated by β_1_ integrin mediated signaling [45] and which is involved in the cerebral cortical development [46] was not altered by alcohol in the diet. This, however, is consistent with the recent findings [15] that shows that the transcripts of cell-cell adhesion molecules ‘Cadherins’ are not altered by alcohol.

Signaling mediated by the integrin receptors is indispensable for the normal development of the brain [47]. It is required for the development of the radial glial fibers and neocortical layers [48]. In the developing cerebral cortex, the expression of β_1_ and α_3_ integrin subunits are detected throughout the cortical wall, whereas the expression of α_6_ subunit is restricted to the ventricular zone and the cortical plate [47]. Both α_3_β_1_ and α_5_β_1_ integrin receptors on neuronal surface are required for the radial migration of neurons [49,50]. Therefore, altered levels of β_1_ and α_3_ integrin subunits and the signaling mediator PLC-γ_2_, caused by gestational alcohol, are expected to change the neuronal positioning causing heteropias [51] and disturb the formation of cerebral-cortical structures.

The peak values of blood alcohol concentrations in the Long-Evans rats (126–155 mg/dL) were in accordance to the values reported earlier with similar diet regimen in this species [10]. Since, the alcohol level in humans on a chronic alcohol diet are known to reach up to 400 mg/dL [52], the altered maternal, fetal and placental weights and the expression levels of molecules in the fetal cerebral cortex observed in this study, indicate that alcohol at half of the concentration achieved during chronic drinking in humans may affect both the health of the mother and the cortical development of the fetus.

## 5. Conclusions

It is well known that human alcoholics become calorie restricted—they eat less as they drink more. Our experiments, by reproducing this effect in an animal model (Figure 2), has more closely fitted a human situation. The absence of obvious developmental defects in the offspring of Long-Evans mothers on chronic alcohol during pregnancy as opposed to other strains of rat [37,53,54] show that the Long-Evans rats may be used as a genetic variant for understanding the mechanisms of neurobehavioral problems that may occur in children in the absences of obvious signs of FAS [55]. Moreover, our study provides the first report of chronic *in vivo* modeling of the effects of alcohol on molecules regulating integrin signaling as previous reports on the changes in the expressions of integrins subunits in fetal brains by alcohol were performed either on brain slices *in vitro* [14] or in an acute *in vivo* exposure model [15]. Future studies are now warranted to dissect the effects of malnutrition and alcohol in pregnant Long-Evans rats. This will be conducted by comparing the results from a group of animals provided with the same volumes of liquid-diet with no alcohol that is consumed by a group of animals on the liquid-diet with alcohol at different gestation days.

In the final analysis, results presented here and those published earlier [14,15,56] demonstrate that integrin-ECM interaction mediated cell signaling is the crucial target of maternal alcohol exposure in the fetal cerebral cortices and may occur in the absence of obvious developmental defects of FAS. Because integrin receptor signaling regulate proliferation and migration of cells in the fetal brains [40,47,48], the prenatal alcohol-mediated changes in the expression levels of molecules involved in integrin receptor signaling would cause wide range of problems during the organogenesis of the fetal brain structures. It is now apparent that inhibiting the effects of prenatal alcohol on the integrin receptor signaling in the fetal brains may reduce the severity of FAS.

## Figures and Tables

**Table 1:** Primary antibodies used for the detection of antigens in the fetal cerebral cortices.

Antigen Primary	Antibody	Source
β-Actin	Mouse monoclonal (Clone C4)	BD Biosciences, San Jose, CA, USA
GPDH	Mouse monoclonal (Clone 6C5)	Millipore corporation, Billerica, MA, USA
Integrin subunit β_1_	Mouse monoclonal (Clone 18)	BD Biosciences, San Jose, CA, USA
Integrin subunit α_3_	Mouse monoclonal (Clone 42/CD49c)	BD Biosciences, San Jose, CA, USA
Integrin subunit α_6_	Rabbit polyclonal (Purified)	ABGENT, SanDiego, CA, USA
Phosphoinositide-specific phospholipaseCγ_1_ (PLCγ_1_)	Rabbit polyclonal (Purified)	Cell Signaling Technologies, Inc., Danvers, MA, USA
Phoshpho PLCγ_1_ (Tyr 783)	Rabbit polyclonal (Purified)	Cell Signaling Technologies, Inc., Danvers, MA, USA
Phosphoinositide-specific phospholipaseC γ_2_ (PLCγ_2_)	Rabbit polyclonal (Q-20) (Purified)	Santa Cruz Biotechnology, Santa Cruz, CA, USA
N-cadherin	Mouse monoclonal (Clone 13A9)	BD Biosciences, San Jose, CA, USA
